# The role of neuropsychological assessment in the investigation of neurodevelopmental disorders

**DOI:** 10.1016/j.jped.2025.101470

**Published:** 2025-11-26

**Authors:** Danielle Irigoyen da Costa, Rosana Vieira de Souza, Thaís Cecília Fiamoncini

**Affiliations:** a Pontifícia Universidade Católica do Rio Grande do Sul (PUCRS), Escola de Ciências da Saúde e da Vida e Escola de Medicina, Porto Alegre, RS, Brazil; b Instituto do Cérebro da PUCRS (InsCer), Porto Alegre, RS, Brazil; c Pontifícia Universidade Católica do Rio Grande do Sul (PUCRS), Programa de Pós-Graduação em Medicina e Ciências da Saúde/Neurociências (PPGMSC), Developmental Cognitive Neuroscience Lab (DCNL), Porto Alegre, RS, Brazil; d Pontifícia Universidade Católica do Rio Grande do Sul (PUCRS), Escola de Ciências da Saúde e da Vida, Porto Alegre, RS, Brazil

**Keywords:** Neurodevelopmental disorders, Neuropsychological assessment, Neuropsychological instruments

## Abstract

**Objective:**

The present study aims to present and discuss the role of neuropsychological assessment (NA) in the investigation of neurodevelopmental disorders (NDDs), with an emphasis on early diagnosis, on the steps that make up the evaluation process, and on the resources that complement clinical evaluation. Furthermore, the discussion is expanded to specific conditions, such as Attention Deficit/Hyperactivity Disorder (ADHD), Autism Spectrum Disorder (ASD), and Specific Learning Disorders (SLD), considering that they represent frequent assessment demands.

**Data sources:**

A critical review of the national and international literature on neuropsychological assessment in NDDs was carried out, highlighting standardized instruments for the Brazilian context, complementary clinical tasks, and main cognitive and behavioral domains. References to essential books and manuals were included, with analysis enriched by the authors' clinical and teaching experience.

**Data synthesis:**

NA constitutes a central tool for the early identification of alterations in neurodevelopment, with contributions to diagnosis and to the definition of intervention strategies. The process involves systematic steps, such as detailed anamnesis, application of standardized tests, and behavior analysis, covering different domains. The feedback, the final stage of the process, is essential for the understanding of the results and for adhering to the suggested procedures, and it should guide parents on practical implications, therapeutic and educational recommendations, and necessary referrals.

**Conclusions:**

NA represents an essential resource in pediatric practice, both for diagnostic support and for directing interventions and monitoring the progress of children with NDDs. Its early use can modify the prognosis and promote better functional, academic, and socioemotional outcomes.

## Introduction

**Neurodevelopment** refers to the continuous process of anatomical and functional maturation of the nervous system (NS), which begins *in utero* and continues more intensely throughout the first years of life. This process involves fundamental stages, such as neuronal proliferation and migration, the formation of synaptic connections, myelination, and synaptic pruning, which enable the progressive acquisition of motor, cognitive, emotional, and social skills [1]. **Neuropsychological development**, on the other hand, results from the dynamic interaction between an individual's genetic heritage, other biological factors, and environmental stimuli, such as family, social, affective, and cultural factors. Thus, early experiences, both positive and adverse, can have a significant impact on brain organization and cognitive and behavioral functioning throughout life.

As a clinical and scientific resource, **neuropsychological assessment** (NA) is highly relevant for understanding the functioning of individuals suspected of having **neurodevelopmental disorders** (NDDs), providing essential information for identifying areas of strength and vulnerability, as well as possible comorbidities. Its main objective is to recognize patterns of functioning (cognitive-adaptive profile) that aid in diagnosis and estimate functional impact, enabling intervention planning and longitudinal monitoring, going beyond the mere description of symptoms and allowing a broader understanding of the patient's profile [2].

The assessment of NDDs should, whenever possible, be conducted by a multidisciplinary team, considering the complexity and variability of clinical manifestations. The integration of different areas, encompassing cognitive, emotional, behavioral, motor, and social aspects, favors not only a more accurate diagnosis but also the development of individualized and effective care/therapeutic strategies aligned with the specific needs of each case.

It is estimated that approximately 250 million children worldwide have some type of neurodevelopmental delay or disability [3]. International data indicate an increasing trend in the prevalence of neurodevelopmental and behavioral disorders within the broader context of disabilities. Among the most prevalent disorders, the following stand out:

• Autism Spectrum Disorder (ASD), with a recent estimate from the Centers for Disease Control and Prevention (CDC) indicating that one in 31 children up to age 8 has been diagnosed with the disorder; [4]
• Attention Deficit Hyperactivity Disorder (ADHD), whose global prevalence is estimated at between 5 % and 8 %, according to the Brazilian Attention Deficit Association (ABDA); [5]
• Specific Learning Disorders (SLD), which affect between 5 % and 15 % of the global population, according to the American Psychiatric Association [6].

According to the World Health Organization [7]. such estimates may be underestimated, considering the difficulties in early diagnosis, the high mortality rates in vulnerable populations, and the stigma associated with these conditions. These are, therefore, highly prevalent conditions whose impact on academic and psychosocial development poses significant challenges throughout the various stages of life. Early diagnosis, combined with appropriate interventions, is essential, as it has a decisive impact on the prognosis and quality of life of children and their families.

## Neurodevelopmental disorders

NDDs constitute a group of clinical conditions that begin during the developmental period, usually in early childhood, and are characterized by deficits in cognitive, behavioral, social, and emotional functioning [6]. These disorders result from alterations in brain development and manifest early, with significant repercussions on the individual's academic, social, and occupational life.

Unlike other mental and behavioral disorders, such as schizophrenia or bipolar disorder, in neurodevelopmental disorders, cognitive and behavioral changes are central manifestations, not secondary or associated conditions [8].

It is important to note that an individual may present comorbidity between NDDs, suggesting the influence of shared genetic and environmental factors. Regarding the etiology, it is known to be complex and multifaceted and, in many cases, unknown.

The Diagnostic and Statistical Manual of Mental Disorders (DSM-5-TR), in its most recent version in Brazil, includes the following NDDs:

• Intellectual Development Disorders
• Communication Disorders
• Autism Spectrum Disorder
• Attention Deficit/Hyperactivity Disorder
• Specific Learning Disorder
• Motor Disorders

## Neuropsychological assessment in the investigation of NDDs

NA is a structured clinical procedure that aims to understand the individual's cognitive, emotional, and behavioral functioning, using standardized tests, neuropsychological tasks, complementary instruments, as well as clinical observations and interviews. There is no fixed battery that is applicable to all cases, since the choice of instruments should be guided by the specific demands of the assessment, the diagnostic hypotheses, and the objectives of the process. Nevertheless, the investigation of certain domains is recommended, such as intellectual functioning, attention and executive functions, memory, language, visuospatial and constructive skills, reading, writing, and math skills, as well as emotional and behavioral aspects (complementary to the cognitive profile) and adaptive functioning (AF). In pediatric practice, NA is used for different purposes:

• **Mapping the cognitive-adaptive profile, aiding in diagnosis**

It consists of describing the neuropsychological profile, identifying the presence or absence of cognitive dysfunctions, and the level of impact on the individual's life [9]. This information aids in diagnosis, as it provides complementary evidence to clinical and contextual data, in addition to guiding appropriate interventions. It would be like constructing a detailed portrait of how the child/adolescent learns, thinks, and adapts to daily life at that moment, allowing the identification of both strengths and vulnerabilities, which guides diagnosis and interventions.

To achieve this goal, various methods and techniques are employed, including interviews, behavioral observations, clinical scales, and the administration of standardized instruments and other neuropsychological tasks to investigate different domains, in addition to qualitative performance analysis [10]. In the case of individuals with NDDs, the diversity of clinical manifestations can make both the selection of the test battery and the interpretation of results an additional challenge [2]. The professional's expertise is essential to define the limits and applicability of each assessment method.

• **Identify the presence of comorbidities and clarify the differential diagnosis** (e.g., distinguish primary manifestations of ADHD from anxiety/sleep effects, etc.)

Neuropsychological assessment, by integrating data from interviews, observation, and the use of standardized instruments, allows us to distinguish primary manifestations of the disorder from those related to comorbidities, contributing to a more accurate diagnosis. Symptoms such as attention difficulties, school problems, or behavioral changes can often be present in different conditions, such as ADHD, SLD, ASD, or anxiety disorders.

• **Estimating functional impact and support needs**

NA is not limited to identifying cognitive deficits; it allows estimating the functional impact of changes observed in the daily life of children with NDD and identifying the most appropriate support needs. Through the integrated analysis of cognitive, emotional, and behavioral skills, combined with qualitative observation and family reports/other sources of information, it is possible to outline needs that will be the focus of intervention and/or educational and social adjustments, promoting the child's functionality and participation in different contexts.

Adaptive functioning (AF) refers to the individual's ability to apply their cognitive skills to daily life, encompassing practical, social, and conceptual aspects. Assessing these domains reveals how the individual copes with the demands of daily life. Domains assessed (according to DSM-5-TR and AAIDD [11]):

(a) **Conceptual**: communication, language, academic skills, reasoning, and memory.
(b) **Social**: interpersonal relationships, empathy, social skills, group participation.
(c) **Practical**: autonomy, self-care, use of community resources, routine organization, safety.

• **Support the planning of interventions and the longitudinal monitoring, as well as guide the family and school**

The results of the assessment have direct implications for clinical and educational management. The information obtained guides individualized intervention programs, pedagogical strategies, and the training of parents and teachers to address the specific demands of each child. The assessment also serves as a baseline for monitoring the evolution of the condition over time and evaluating the effectiveness of the implemented interventions.

### Stages of the assessment process

To assist in understanding this systematic and rigorous process, eight steps are highlighted, suggested by other authors [1] and here adapted to the Brazilian context, which must be integrated to record the information provided and appropriately interpret and communicate the results: [1]

(1) **Attentive listening to the reason for seeking consultation:** This initial phase focuses on understanding the reported concerns and needs, considering the factors involved, the affected individuals, and the manifestations of the condition in the patient and their environment. It is an opportunity for parents/caregivers and children to feel heard and understood, creating a comfortable environment for the following steps. According to the Code of Professional Ethics for Psychologists ( Art. 8) [12] and Federal Council of Psychology (Resolution No. 13/2022) [13], which consolidates standards and principles for the provision of psychological services to children and adolescents, conducting a neuropsychological assessment in children requires formal authorization from at least one legal guardian.
(2) **Knowledge of the child's clinical and academic history, behavior and family background:** Includes personal data (name, date of birth, chronological age, school year, school, with whom the child lives), clinical history (neuropsychomotor development, chronic conditions, medical treatments and pharmacological and non-pharmacological interventions), academic history (age of entry into school, performance, learning problems, failures, adaptation, changes of school), behavior (relevant traits, sociability, anxiety, mood, motivation, sleep disorders) and family history (home environment, age and level of education of parents and siblings, family history of NDDs). It is crucial to delve deeper into the child's AF at this stage [1].
(3) **Analysis of complementary examinations and of additional health and educational records:** This stage involves reviewing previous neuropsychological evaluations, reports from teachers and/or other professionals working with the child, and relevant medical examinations [1]. It also considers the presence of curricular adaptations, specialized educational service plans, tutoring interventions, or other relevant educational support strategies.
(4) **Permanent clinical observation:** This includes the child's initial contact (visual, posture, communication), participation and engagement during the interview and testing/task completion, motivation, energy level, state of consciousness, attention, comprehension, and mood. It considers possible interferences such as changes in vigilance, attention, and energy due to lack of sleep and/or medication use, allowing for the optimal timing of the evaluation.
(5) **Application of neuropsychological tests and neuropsychiatric scales:** These are highly relevant assessment tools. Tests/tasks and scales appropriate for the specific clinical case should be selected, with known psychometric properties. Standardizing these tests ensures the validity and reliability of the data.
(6) **Emotional aspects assessment:** Although not essentially part of neuropsychological studies, it is recommended to include it, as emotional changes are a frequent comorbidity in NDDs and can interfere with cognitive processes [1]. Understanding socioemotional factors helps identify the origin of a certain behavior and inform the planning of more effective interventions. Neuropsychological repercussions in children can impact the school, family, and social environments, leading to interaction difficulties and influencing self-image. For assessment, the use of instruments such as depression and anxiety questionnaires, self-concept scales, and measures of socioemotional skills, among others, is recommended.
(7) **Analysis of the child's results:** The child's performance, expressed in standardized scores for their chronological age or school year, is analyzed considering the reason for the consultation, clinical observations, diagnostic hypothesis, possible comorbidities, and the context in which the child develops. Qualitative analysis in NA is also important, as it complements the results of objective tests and helps to better understand the performance of the person being evaluated. Finally, a report is prepared that integrates all this information, providing a comprehensive view of the child's condition and the suggested actions based on the findings.
(8) **Delivery of results to interested parties (feedback):** This includes legal guardians and the child themselves (considering their age, maturity, and level of understanding). Feedback can also be provided to members of the school system and to the health and education professionals involved, with written consent from the legal guardians. Even with authorization, the psychologist must ensure that the information shared is relevant, ethical, and to the extent necessary, preserving confidentiality and avoids undue exposure of the child.

Emphasis is placed on the analysis of the neuropsychological profile, its “translation” into school, home, and social life, and recommendations for necessary interventions and support. The feedback interview is a fundamental step in the neuropsychological assessment, as it allows clear communication of findings, guides interventions, and supports families, ensuring engagement in the suggested behaviors and ethical monitoring of the child.

It is important to note that the neuropsychological profile is dynamic and can vary over time due to the maturation process and therapeutic and pedagogical efforts. Furthermore, most traditional tests are standardized on typically developing populations and may not consider crucial factors in patients with NDDs, such as fatigue, boredom, low self-esteem, difficulties with motor control, understanding instructions, and stress. Therefore, clinical judgment and the development of new tools are essential for a more accurate assessment.

## Cognitive and behavioral assessment tools in NDDs

Among the main domains investigated are: intelligence, attention and executive functions, memory, oral language, reading and writing, visuospatial and visuoconstructive functions, as well as socioemotional skills, other behavioral aspects, and AF. As previously mentioned, there is no single battery capable of assessing all NDDs; the choice of instruments depends on the specific objective of the investigation, the individual's overall level of functioning, and medication use, among other factors. The examiner's expertise is crucial, as they have the autonomy to select the most appropriate instruments to answer the clinical questions presented.

The combined analysis of these elements allows the construction of a profile of strengths and weaknesses, essential for understanding each individual's uniqueness [14].

Table 1 [15, 16, 17, 18, 19, 20, 21, 22, 23, 24, 25, 26, 27, 28, 29, 30, 31, 32, 33, 34, 35, 36, 37, 38, 39, 40, 41, 42, 43, 44, 45, 46, 47, 48, 49, 50, 51, 52, 53, 54, 55] presents the main instruments currently used in neuropsychological practice in Brazil, indicating the investigated domains. It is noteworthy that, although each neuropsychological instrument preferentially assesses a specific construct, they involve different skills. Therefore, the examiner's experience is a key differentiator in integrating the obtained data, based on clinical reasoning, to guide the best course of action at the end of the investigative process [56]. It should also be noted that neuropsychological assessment is a complex process that goes beyond the mere application of tests and tasks/other scales, and always involves a series of steps, in addition to the integration of knowledge necessary for the clinical context (neuroanatomy, psychopathology, child development, influences of the socioeconomic context, psychometrics, among others).

**Table 1:** Neuropsychological Tests and Tasks and Instruments for Behavioral Assessment in NDDs.

DOMAIN	INSTRUMENT	BRIEF DESCRIPTION
**Intelligence**	WECHSLER INTELLIGENCE SCALE FOR CHILDREN - WISC-IV(16)	This is a standardized psychological test designed to assess the intellectual performance of children and adolescents. It provides the **overall Intelligence Quotient (IQ)** as well as four main indices: • **Verbal Comprehension (VC)** • **Working Memory (WM)** • **Perceptual Organization (PO)** • **Processing Speed (PS)** The instrument identifies strengths and areas of vulnerability and is widely used in clinical, educational, and neuropsychological settings, including the diagnosis of neurodevelopmental disorders. It is recommended for children and adolescents aged **6 to 16 years and 11 months.**
	WECHSLER ABBREVIATED SCALE OF INTELLIGENCE -WASI(15)	It is an instrument developed to meet the need for a rapid assessment of intelligence, presenting validity and accuracy suitable for use in clinical and research contexts. It provides scores of **Verbal IQ, Performance IQ, and Total IQ** . The scale **is composed of four subtests** — Vocabulary, Cubes, Similarities and Matrix Reasoning — and allows a brief estimate of general cognitive functioning, both verbal and non-verbal. It is indicated for individuals aged **6 to 89 years old**.
	SNIJDERS-OOMEN NON-VERBAL INTELLIGENCE TEST -SON-R 2½−7[a](17)	It consists of a non-verbal intelligence test that assesses general cognitive development and abilities, focusing on **spatial, visual-motor and reasoning abilities.** Because it doesn't require language, it's recommended for children with communication difficulties, such as deafness and neurodevelopmental disorders, and is also useful for immigrants. It can be applied in clinical, educational, and neuropsychological settings. Suitable for children from **2 years and 6 months to 7 years and 11 months old.**
	RAVEN'S COLORED PROGRESSIVE MATRICES(18) - CPM	It is considered the gold standard for assessing general intelligence (g Factor), an **educational skill** linked to the ability to generate new insights in nonverbal situations. Quick and engaging to administer, with colorful stimuli and minimal reading requirements, it is recommended for children with language difficulties, physical disabilities, aphasia, cerebral palsy, deafness, intellectual impairment, and NDDs. It can be used in clinical, educational, and research settings. Suitable for children between **5 and 11 years and 11 months old**.
**Attention and Executive Functions**	FIVE DIGITS TEST – FDT(19)	It is a brief instrument that assesses **processing speed, attention, and executive functions** (inhibitory control and cognitive flexibility). It consists of **four parts**: Reading and Counting (automatic and simple processes), and Choosing and Switching (more complex processes that require active mental control). Recommended for children from **6 years of age to the elderly**.
	PSYCHOLOGICAL BATTERY FOR ATTENTION ASSESSMENT-2 (BPA-2)(20)	Its purpose is to measure **overall attention span**, as well as assess specific types of attention: **concentrated, divided, and alternating attention**. Indicated for people aged **6 to 94**.
	TRAIL MAKING TEST FOR PRESCHOOLERS(21)	**The Trail Making Test for Preschoolers** aims to assess **cognitive flexibility**, i.e., the ability to switch between different tasks or rules efficiently. The test is administered briefly, allowing a quick analysis of the child's performance on tasks that require **attention, planning, and adaptation to change**. It is especially useful in **neuropsychological assessment and cognitive development** contexts, providing information on early executive skills and helping to identify possible cognitive or attention difficulties. **Indicated for children aged 4 to 6**.
	WISCONSIN CARD SORTING TEST - WCST(22)	The **WCST** assesses **executive functions**, including strategic planning, organization, impulse control, and adaptation to environmental changes. It measures the ability to **develop strategies to solve problems and achieve goals** and is useful for identifying **cognitive impairments and neurological changes related to the frontal region of the brain.** Indicated for children aged **6 years and 6 months to 89 years and 11 months.**
	VISUAL ATTENTION TEST – 4TH EDITION (TAVIS-4)(23)	The **TAVIS-4** is a computerized test that assesses attentional functions in children and adolescents. It measures **selective, sustained, and alternating attention** through visual tasks. The main performance indicators are **average reaction time, errors of omission, and errors of action**. The administration is individual and lasts between 12 and 20 min, depending on age. Brazilian standards are available, allowing interpretation of performance in relation to age and gender. Indicated for children and adolescents **aged 6 to 17**.
	STROOP (VICTORIA VERSION)(24)	The Stroop Test assesses **selective attention and processing speed**, assessing an individual's ability to classify information from the environment and respond in a controlled manner to conflicting stimuli. Indicated for individuals **aged 7 to 10** (standards of the cited study).
	VERBAL FLUENCY - VF (25)	VF tests were initially introduced to assess frontal lobe lesions in adult patients, and their study was later extended to the neuropsychological evaluation of children and adolescents. They are widely used in neuropsychological assessments because they are quick and easy to administer and involve the individual recalling words for a specific period, typically 60 s. In clinical practice, the most commonly used VF tests are **Semantic Verbal Fluency** (SVF) and **Phonemic Verbal Fluency** (PVF). In Brazil, there are studies for preschoolers, school-age children, as well as adolescents, adults and the elderly.
**Memory**	REY AUDITORY VERBAL LEARNING TEST - RAVLT(26)	The **RAVLT** assesses **episodic declarative memory**, providing measures of **verbal learning, retention, recognition, and resistance to interference**. It allows the development of a **verbal learning profile** and the analysis of memory **encoding, storage, and retrieval** processes, making it a relevant tool for **neuropsychological diagnosis and assessment.** Indicated for individuals **aged 6 to 92.**
	COMPLEX FIGURES OF REY: COPYING AND MEMORY REPRODUCTION TEST OF COMPLEX GEOMETRIC FIGURES(27)	Rey Complex Figures assess **visual perception** and **immediate memory** through two phases: **memory copying and memory reproduction**. The test investigates how individuals grasp visual information and what they can retain spontaneously. Indicated for children **ages 4 and older** (standards in Brazil are being updated).
**Language**	CHILDREN'S ORAL NARRATIVE DISCOURSE (DNOI)(28)	The DNOI is a structured task that assesses children's ability to produce oral narratives. Performance is analyzed based on criteria such as fluency, temporal organization, and use of connectives. This instrument is useful for identifying difficulties in children with language and learning disorders. Recommended for children **aged 6 to 12**.
	CHILD LANGUAGE TEST - ABFW(29)	The **ABFW** is a language test comprised of four tasks (Phonology, Vocabulary, Fluency, and Pragmatics), used both in **initial speech-language pathology assessments** and in **monitoring child development**. Adapted to the **Brazilian linguistic and cultural context**, it offers **psychometric reliability and accuracy,** making it an evidence-based tool for professional practice. Indicated for children **aged 2 to 12**.
	PEABODY PICTURE VOCABULARY TEST - PPVT(30)	The **receptive vocabulary assessment** measures the ability to understand words, providing useful information about **language development** throughout life. It allows the identification of **strengths and weaknesses in the semantic domain** (word knowledge), aiding in the overall analysis of **language skills** and the planning of appropriate interventions. Indicated for children aged **2 years and older**.
**Reading and Writing**	SCHOOL PERFORMANCE TEST II - TDE-II(31)	The **TDE** II assesses learning in reading, writing, and mathematics throughout the **nine years of elementary school**, and is useful in contexts of both **typical and atypical development**. It allows the mapping of academic skills and the selection of **more effective intervention strategies** in **clinical and educational** settings. It is recommended for **students in grades 1 to 9**, which corresponds, on average, to the **age range of 6 to 14 years**.
	READING PROCESS ASSESSMENT TESTS -PROLEC(32)	It is a comprehensive instrument for assessing **reading and textual comprehension in children,** widely used in clinical and school settings in Brazil. It is recommended for children **aged 6 to 12**, covering the 2nd to 5th grade of elementary school.
	CONFIAS(33)	**CONFIAS** It is a test that assesses **phonological awareness** comprehensively and sequentially, covering skills such as **syllables, rhymes, alliteration, intrasyllabic units, and phonemes.** It consists of tasks involving **synthesis, segmentation, identification, production, exclusion, and syllabic and phonemic transposition**, divided into two parts: **syllable awareness** and **phoneme awareness**. The instrument is recommended for children who are **illiterate or in the process of learning to read**, as well as for **working with learning difficulties or disorders**. Suitable for children **ages 4 and older**.
	NEUROPSYCHOLOGICAL EVALUATION OF READING AND WRITING - ANELE(34–38)	**ANELE** (Neuropsychological Assessment of Reading and Writing) is a collection of psychometric instruments developed to assess reading and writing skills in children, adolescents, and adults, based on theoretical models of cognitive neuropsychology and psycholinguistics. The collection consists of five volumes, each focusing on specific aspects of reading and writing.
**Visuospatial and Visuoconstruction Functions**	FREE DRAWING TESTS OR COPYING OF GEOMETRIC SHAPES (16,39)	In clinical and neuropsychological contexts, they allow qualitative assessment of visuoconstruction.
	BENTON VISUAL RETENTION TEST - BVRT(40)	In Brazil, the **Benton** is used both in a **standardized and qualitative way**, especially when it comes to understanding **visual-constructive and visual memory profiles**. It is normally applied to **children aged 7 and older**, adolescents, and adults, but adaptations are available for younger age groups.
Behavior, socioemotional aspects and neuropsychiatric manifestations*	SOCIAL RESPONSIVENESS SCALE – 2ND EDITION (SRS-2)(41)	It identifies a wide range of impairments in reciprocal social behavior (from absent to severe) based on observation of an individual's behavior in naturalistic social contexts and classifies them as mild, moderate, or severe. The SRS-2 (Brazilian version) can be administered to preschool children aged **2.5 to 4.5 years**, schoolchildren aged **4 to 18 years**, and adults aged **18 years and older**, allowing the assessment of social responsiveness across different age groups.
	MODIFIED CHECKLIST FOR AUTISM IN TODDLERS, REVISED WITH FOLLOW-UP - M-CHAT-R/F(42)	O M-CHAT-R/F is a two-stage, parent-reported screening tool for assessing the risk of **Autism Spectrum Disorder (ASD)** in children aged 16 to 30 months. The first stage consists of 20 "yes" or "no" questions, and the second stage is a follow-up interview to clarify ambiguous or moderate-risk responses.
	CHILDHOOD AUTISM RATING SCALE (CARS)(43)	It consists of 15 items, each rated on a scale of 1 to 4, that reflect typical autistic behaviors. Its administration involves direct observation of the child and input from caregivers. It is recommended for children **aged 2 and older** to identify the presence and severity of **ASD** symptoms in this age group. In the Brazilian study cited (CARS-BR), the age of the participants ranged from 3 to 17 years.
	AUTISM DIAGNOSTIC INTERVIEW-REVISED (ADI-R)(44)	It is a structured interview instrument for parents or caregivers, used to aid in the **diagnosis of ASD**. Suitable for children and adolescents **aged 2 and older**.
	AUTISM DIAGNOSTIC OBSERVATION SCHEDULE - ADOS(45)	Structured interview and observation activities that assess communication, social interaction, play/imagination, and restricted and repetitive behaviors. It requires formal training to administer, usually by psychologists, psychiatrists, or pediatric neurologists. It complements interviews with family members (such as the ADI-R) and aids in the diagnostic confirmation of ASD, treatment planning, and research. Used for children from **18 months** to adulthood, with five modules that adapt to the individual's language ability and age.
	EVALUATION PROTOCOL FOR CHILDREN WITH SUSPECTED AUTISM SPECTRUM DISORDERS – REVISED VERSION (PROTEA-R)(46)	This instrument was developed in Brazil to screen for early signs of **ASD**. It is recommended for **children aged approximately 2 to 5 years,** focusing on nonverbal children or those with limited communication skills. The instrument is particularly useful for identifying early signs of ASD and other communication disorders, enabling early intervention. It is widely used by professionals such as psychologists, speech-language pathologists, occupational therapists, and physicians, and is applicable in clinical, educational, and research settings.
	CHILD BEHAVIOR CHECKLIST - CBCL(47)	**CBCL** It is a reliable instrument for **assessing behavioral and socioemotional functioning** in children and adolescents, widely used in clinical and academic settings. It allows for monitoring changes in behavior over time. In the Brazilian version, it is intended for children and adolescents **aged 6 to 18**, covering the entire elementary and high school period.
	SNAP-IV(48)	This questionnaire is widely used to assess symptoms of **Attention-Deficit/Hyperactivity Disorder (ADHD)** and **Oppositional Defiant Disorder (ODD)** in children and adolescents. It can be completed by **parents and teachers**, allowing observation of the individual's behaviors in **different contexts,** such as home and school. In addition to aiding in **screening and diagnosis**, the instrument is useful for **monitoring changes over time** and supporting the planning of **educational and clinical interventions**. **Indicated for children and adolescents aged 6 to 18.**
	ATTENTION DEFICIT/HYPERACTIVITY DISORDER SCALE IN SCHOOL CONTEXT – VERSION FOR TEACHERS - ETDAH-II(49)	The ETDAH-II is a **teacher-administered** instrument used to identify **ADHD** symptoms in the school setting. It assesses attention, hyperactivity/impulsivity, and **potential impairments in academic performance, school skills, and social functioning**, contributing to monitoring and intervention planning. Indicated for children and adolescents between **6 and 18 years of age.**
	SCALE FOR ASSESSING CHILDREN'S BEHAVIORS IN ATTENTION DEFICIT/HYPERACTIVITY DISORDER IN THE FAMILY ENVIRONMENT – VERSION FOR PARENTS ETDAH (50)	The ETDAH-PARENTS is an instrument administered to parents to assess the behavior of children and adolescents in the home environment. It identifies symptoms of **attention, hyperactivity, and impulsivity,** as well as **emotional regulation and adaptive behavior**. Indicated for children and adolescents between **2 and 17 years** of age.
**Adaptation and Functionality**	VINELAND-3 ADAPTIVE SCALE (51)	**Vineland-3** is a tool used to assess the **adaptive behavior** of individuals from birth. It consists of a semi-structured interview in a questionnaire format, allowing the assessment of the adaptive behavior of children, adolescents, young adults, and adults through information provided by third parties, such as parents, caregivers, and teachers. The main domains assessed include Communication, Daily Skills, Socialization, Motor Skills, and Maladaptive Behaviors.
	ADAPTIVE FUNCTIONING SCALE (EFA)(52)	The **Adaptive Functioning Scale (EFA)** is an assessment instrument aimed at **children and adolescents between the ages of 6 and 15**. It is a Brazilian instrument developed based on normative studies covering all regions of the country. It assesses the three domains of Adaptive Functioning, according to **DSM-5** criteria, allowing an accurate and reliable analysis.
	ADAPTIVE BEHAVIOR ASSESSMENT SCALE – THIRD EDITION (ABAS-3)(53)	ABAS-3 was translated and culturally adapted into Brazilian Portuguese to assess the adaptive skills of individuals aged **0 to 89**. The scale covers three main domains: conceptual, social, and practical, and consists of forms intended for parents, teachers, and professionals.
**Neuropsychological Batteries**	BRIEF NEUROPSYCHOLOGICAL ASSESSMENT INSTRUMENT NEUPSILIN-INF(54)	**NEUPSILIN** is a Brazilian neuropsychological battery intended for children **aged 6 to 12**, designed to assess cognitive functions such as **attention, memory, language, executive functions, visuospatial skills and praxis**.
	NEPSY-II(55)	**NEPSY-II** is a neuropsychological battery for children aged **3 to 16**, designed to assess cognitive functions essential for child development. Its domains include **attention and executive functions, language, memory and learning, social perception, sensorimotor functions, and visuospatial processing**. Each domain has specific subtests that allow an assessment of cognitive abilities, being useful for diagnosis, intervention, and developmental monitoring.

* Some instruments are specific to certain neurodevelopmental disorders, while others have cross-sectional application and can be used in different conditions to assess cognitive, behavioral, or socioemotional domains.

For infants and young children, **specific developmental assessment scales** are generally used, as detailed in Table 2, [57, 58, 59] allowing the early identification of skills and possible delays in different domains of neuropsychomotor, cognitive, and socioemotional growth.

**Table 2:** Scales for assessing child development.

**Development assessment**	DIMENSIONAL INVENTORY FOR ASSESSING CHILD DEVELOPMENT - IDADI AND IDADI- BRIEF(57)	It assesses the seven domains of child development: cognitive, socioemotional, receptive and expressive language, gross and fine motor skills, and adaptive behavior. Parents of children aged **4 to 72 months**.
	DEVELOPMENTAL SCREENING TEST - DENVER II(58)	It also provides information for planning interventions. It assesses developmental progress across four domains: personal-social, fine-motor-adaptive, gross-motor, and language. **Indicated for children ages 0 to 6**.
	BAYLEY SCALES OF INFANT AND TODDLER DEVELOPMENT, THIRD EDITION - BAYLEY III(59)	It identifies developmental delays across five domains: cognitive, linguistic, motor, socio-emotional, and adaptive behavior. **Indicated for infants aged 1 to 42 months**.

## Applications in specific disorders (ADHD, ASD, and SLD)

In clinical practice, neuropsychological assessment has wide application in different NDDs. Generally speaking, in cases of ADHD, it can be said that it helps characterize the predominant type of symptoms and their functional impact. SLD allows us to identify which reading, writing, or math skills are most compromised, guiding targeted pedagogical interventions. In ASD, it helps to map preserved cognitive skills and associated deficits, aiding in the definition of teaching and support strategies. Thus, the discussion focused on specific conditions, presented later, considering that they represent frequent assessment demands.

## Neuropsychological assessment in ADHD: points of attention for clinical practice

The diagnosis of ADHD is essentially clinical, based on DSM-5-TR and/or ICD-11 criteria and information obtained from multiple sources. Although the NA is not mandatory, international guidelines, such as those of the American Academy of Pediatrics (AAP), recommend its use in specific situations, such as in cases of diagnostic uncertainty, the presence of comorbidities, or the need for detailed characterization of the cognitive and functional profile [60].

Thus, the NA plays a relevant role in ADHD, contributing to the understanding of the different manifestations of the disorder, characterizing severity and prognosis, and identifying comorbidities, as mentioned above. Structured or semi-structured interviews, symptom screening scales, and other instruments that aid in the identification of psychiatric symptoms can be used for this purpose, in addition to instruments for cognitive and behavioral assessment (such as those described in Table 1), clinical observations, and informant reports. The exam is also useful in defining cognitive therapeutic targets, which can be addressed through both pharmacological and non-pharmacological interventions [61].

It is important to emphasize that **there is no universal assessment protocol for ADHD:** it should be planned based on the patient's history, considering age, potential comorbidities, medication use and their effects, as well as an estimate of overall cognitive level (61). Therefore, the neuropsychologist must be familiar with theoretical models of cognitive functioning, remembering that assessment goes far beyond the administration of tests and tasks. The analysis of results should always consider environmental, contextual, and cultural factors.

Although there is heterogeneity, some patterns are frequently observed in children with ADHD:

• **Attention**: impaired sustained and selective attention, with increased vulnerability to distraction.
• **Executive functions**: difficulties with response inhibition, planning, decision-making, cognitive flexibility, and performance monitoring.
• **Working memory**: deficits in retaining and manipulating information, affecting reasoning and learning.
• **Processing speed**: slowing down in tasks that require speed and precision (often as a means of achieving greater accuracy in performance).
• **Inhibitory control**: difficulties in inhibiting automatic/impulsive responses.
• **Emotional regulation**: difficulties in modulating emotions, often related to low frustration tolerance.
• **Cognitive and Behavioral Self-Regulation**: Persistence, mental effort, monitoring performance and time.

It is important to emphasize, however, that caution is needed when interpreting the results, as a significant portion of patients with ADHD may perform within normal limits on traditional neuropsychological tests [61]. The opposite is also true; excessive agitation may be associated with, for example, psychomotor overexcitability in high ability /giftedness (HA/G), anxiety, and other disorders, and is not “synonymous” with ADHD with a predominantly hyperactive/impulsive presentation.

## Neuropsychological assessment in ASD: points of attention for clinical practice

Due to the complexity and heterogeneity of the neuropsychological profile of ASD, the term "spectrum" reflects the wide variability of cognitive, behavioral, and adaptive manifestations among individuals. Each person may show distinct combinations of abilities and challenges, which require a comprehensive and individualized assessment, covering domains such as attention, memory, executive functions, language, visuospatial and socioemotional skills, sensory processing, and emotional regulation. In this context, the use of standardized and targeted scales (suggested in Table 1) can optimize assessment time and provide objective data on characteristics essential for diagnosis, enabling the identification of behavioral and cognitive patterns typical of ASD, but always in conjunction with clinical practice (instruments are part of the process, but clinical judgment always prevails). Integrating data from family members, teachers, and others (multiple sources/different contexts) and clinical observations allows not only the identification of deficits but also the recognition of specific strengths and talents, providing support for the planning of personalized interventions aimed at maximizing functional potential and promoting quality of life.

At the end of the assessment, suggested actions should include referrals to appropriate services and interventions (and therapeutic options), as well as guidance to the family on the diagnosis, legal rights, and educational and social resources, aiming to support the individual's development, inclusion, and quality of life.

In the ASD NA, the authors emphasize 10 points that deserve special attention to ensure the assessment is clinical, ethical, and effective (illustrated in Table 3).

**Table 3:** 10 points of attention for neuropsychological assessment in ASD.

#	Point of Attention for clinical practice	Description
1	**Level of global development**	Consider chronological age, cognitive level and adaptive profile
2	**Associated comorbidities**	ADHD, anxiety, epilepsy, intellectual disability, among others
3	**Language skills**	Adaptations for verbal and nonverbal communication difficulties
4	**Flexibility and restricted interest**	Be aware of how reduced flexibility and restricted interests can impact task engagement and choice of instrument.
5	**Sensory sensitivity**	Avoid auditory, visual or tactile overload during the exam
6	**Motivation and engagement**	Use of reinforcers, breaks, and adjusting session length: In the assessment of children with ASD, reinforcers are used to maintain attention, motivation, and reduce anxiety during tasks. They can be social (praise), tangible (toys, stickers), sensory (sounds, textures), or preferred activities. They should be individualized and planned so as not to interfere with the results. The evaluator monitors their effect to ensure that performance reflects actual abilities.
7	**Validity of instruments**	Prioritize tests adapted and validated for the Brazilian population
8	**Complementary methods**	Integrate scales, inventories, clinical interviews, and parent/teacher reports
9	**Application context**	Structured, predictable, low-stimulus environment
10	**Interpretation of results**	Consider the heterogeneity of the spectrum and do not restrict the assessment to deficits

## Neuropsychological assessment in SLD: points of attention for clinical practice

SLDs, which include dyslexia, dyscalculia, and dysgraphia, are characterized by significant and persistent impairments in specific academic skills that cannot be explained by sensory or intellectual deficits, or lack of schooling. The literature shows that these disorders are often associated with deficits in core neuropsychological functions, such as phonological awareness, working memory, sustained and selective attention, processing speed, receptive and expressive language, in addition to executive functions such as inhibition and cognitive flexibility. Another clinical indicator is avoidance of activities that require academic skills.

Children with dyslexia may demonstrate significant impairments in verbal working memory and phonological processing, while those with dyscalculia have more marked impairments in visuospatial working memory and the mental representation of quantity [62]. Transdiagnostic studies point to three essential cognitive pillars—phonological processing, processing speed, and executive functions—that significantly explain academic difficulties in reading and mathematics, regardless of the presence of ADHD [63].

Neuropsychological assessment plays an essential role in identifying and characterizing these impairments, using instruments that accurately assess the relevant cognitive domains. In addition to quantitative analysis of scores, the assessment also relies on qualitative data, such as compensatory strategies, error type, and performance pattern, which are essential for the differential diagnosis between disorders and secondary school difficulties. Studies show that children with SLD perform worse on memory tasks, executive functions, and perceptual-motor skills when compared to peers with typical development, reinforcing the importance of a comprehensive assessment that is sensitive to the child's neurocognitive profile [64].

The interpretation should also consider contextual factors, schooling/access to stimulation, and socioemotional aspects. Collaboration with speech-language pathologists, educational psychologists, and teachers is essential for an effective, evidence-based treatment plan.

## Conclusion

NA plays a central role in the investigation of NDDs, allowing not only a better diagnostic definition but also providing concrete support for individualized interventions and the building of support networks between family, school, and health services. Given the inherent complexity of these conditions, the need for a rigorous, multifaceted, and culturally sensitive approach is highlighted, integrating standardized psychometric instruments, qualitative observations, and the work of multidisciplinary teams as key elements for an accurate diagnosis and an effective intervention plan. Furthermore, constant updating of international classifications and validation of instruments for the Brazilian population are essential steps toward improving the quality of care for individuals with NDDs.

Early diagnosis is essential to minimize the impact on a child's overall development. Although each disorder has specific clinical manifestations, many share comorbidities and symptomatic overlap, requiring an integrated approach to assessment and treatment. Furthermore, the recognition of neurological diversity has fostered significant advances in how these disorders are understood, treated, and included in society.

However, it is crucial to recognize existing limitations. Available instruments are not always sufficiently sensitive to the cultural and socioeconomic specificities of the Brazilian population, requiring professionals to carefully analyze and contextualize results when interpreting them. This is compounded by the challenge of access, as many families still face significant barriers to accessing specialized services. Despite these challenges, future prospects are promising. Technological advances, such as the application of computerized tests and the use of artificial intelligence, tend to increase the accuracy and accessibility of assessments. Integration with neuroimaging and genetic data may also offer a more comprehensive understanding of NDDs.

## Financial support

None declared.

## Research data availability

The full dataset supporting the findings of this study is available upon request to the corresponding author.

## Conflicts of interest

The authors declare no conflicts of interest.
